# The neuropsychology of healthy aging: the positive context of the University of the Third Age during the COVID-19 pandemic

**DOI:** 10.1038/s41598-023-33513-4

**Published:** 2023-04-19

**Authors:** Martina Amanzio, Giuseppina Elena Cipriani, Massimo Bartoli, Nicola Canessa, Francesca Borghesi, Alice Chirico, Pietro Cipresso

**Affiliations:** 1 Department of Psychology, University of Turin, 10024 Turin, Italy; 2 ICoN Center, Scuola Universitaria Superiore IUSS, 27100 Pavia, Italy; 3 Istituti Clinici Scientifici Maugeri IRCCS, Cognitive Neuroscience Laboratory of Pavia Institute, 27100 Pavia, Italy; 4 Department of Psychology, Research Center in Communication Psychology, Università Cattolica del Sacro Cuore, 20123 Milan, Italy; 5 Istituto Auxologico Italiano, IRCCS, 20145 Milan, Italy

**Keywords:** Human behaviour, Cognitive ageing

## Abstract

Older adults have been reported to have increased susceptibility to the adverse effects of SARS-CoV-2 infection, such as fatal outcomes, cognitive decline, and changes in physical and/or mental health. However, few studies have examined neuropsychological changes by comparing measurements before and during the pandemic in healthy older people. In addition, no longitudinal studies have examined whether older adults may have responded positively to the pandemic. We examined these issues through a 2-year neuropsychological study before and during the pandemic period. Results showed that scores before and during the pandemic were the same in memory and attention, whereas global cognitive, executive, and language functions improved. Participants also showed no longitudinal changes in depression, hypomania, and disinhibition, while apathy and, to a lesser extent, anxiety increased significantly. To examine possible signs of pandemic-related emotional (dys)regulation, subjects were shown images at follow-up that recalled the most dramatic lockdown phase while heart rate variability was recorded. Higher apathy was predicted by poorer global cognitive performance, increased anxiety, and emotional dysregulation as measured by a higher ratio of low-to-high frequency heart rate variability. Thus, preserved global cognition appears to play a protective role against the effects of pandemic-related anxiety and emotional dysregulation on apathy.

## Introduction

The COVID-19 pandemic had a significant impact on older adults, as they were more vulnerable to both the adverse effects of SARS-CoV-2 contagion^1^ and the measures taken by governments to contain the spread of infection^2^. While the infection curve has been effectively contained, restrictive measures have increased social isolation and loneliness^3^, which in turn affects well-being and increases physical frailty, cognitive impairment, and mood swings^4^. Nursing home residents and patients with Alzheimer's disease or other dementias are those most likely to suffer the effects of the COVID-19 pandemic^4,5^, placing them at highest risk of a fatal outcome or long COVID-19 impact^6^.

A substantial amount of data collected on older adults during the pandemic came mostly from online cross-sectional surveys. For example, self-reported data from an online survey were used to examine the impact of the COVID-19 period on well-being, activity levels, sleep quality, and cognitive function^7^. These studies allowed for large samples to be analyzed, but with notable limitations in terms of the generalizability of the results. First, the fact that not all older adults could be reached via online technologies suggests possible biases in the recruitment of the sample. In addition, the use of self-report questionnaires instead of in-depth neuropsychological assessments made it difficult to clearly classify participants as healthy or cognitively impaired. Finally, the lack of longitudinal studies with neuropsychological data collected before the pandemic made it impossible to detect potential changes in cognitive functioning and/or mood, limiting the resulting findings to short-term effects and neglecting the crucial role of "baseline" performance.

Indeed, in assessing the multifaceted consequences of the COVID-19 pandemic, it is important to consider that cognitive decline may be part of normal aging (e.g.^8–10^), in addition to a gradual decline in physical abilities that may limit functional abilities of daily living and quality of life (e.g.^11^). However, little is known about the impact of the COVID-19 pandemic on healthy aging in individuals who reported not being infected with SARS-CoV-2.

Taking advantage of available pre-pandemic measurements, it has been previously reported longitudinal evidence of early neuropsychological changes during the pandemic period in healthy older individuals^12,13^. A longitudinal study first examined the role of neuropsychogeriatric factors in lockdown fatigue, by comparing data collected in healthy, cognitively aging, individuals before and during the pandemic. Participants were assessed at three different time points during the pandemic, i.e., during the first lockdown period (T1), immediately afterward (T2), and during the second lockdown period (T3). Results highlighted interacting changes of physical functioning, executive attention, and mood deflactions in the COVID-19 pandemic. Subjects with moderate fatigue reported more depressive and anxious symptoms than subjects with mild fatigue. Cognitive performance in terms of psychomotor speed also appears to play an important role in the perception of fatigue associated with COVID-19 restrictive measures. Specifically, the results of principal component and multiple regression analyses demonstrated the contribution of "cognitive" and "psychological" factors (i.e., attentional and executive performance, as well as mood deflections) in explaining handgrip strength and gait speed as two of five determinants of the Fried frailty model^14^. At T3, lockdown fatigue was explained by higher scores on the Beck Depression Inventory and lower performance on the Trail Making Test Part A. The results of a moderated mediation model showed that the effect of psychomotor speed on lockdown fatigue was mediated by depression, with gait speed having a moderating effect on this relationship^12^.

Furthermore, fear of infection could be an additional source of concern for this at-risk population, exacerbating anxiety and thus affecting quality of life^15^. Consistent with this hypothesis, we have previously reported longitudinal data demonstrating that perceived threat associated with the consequences of SARS-CoV-2 infection was predicted by a combination of baseline physical, cognitive, and mood measures, i.e., anxiety and frailty in addition to lower information processing speed and language comprehension performance^13^.

To date, however, no longitudinal study has examined whether healthy older people may also have responded to the COVID-19 pandemic with improvements in neuropsychological functioning. This important but under-researched topic could shed light on how the cognitive and functional abilities that enable the well-being of older subjects are maintained during a very difficult time such as a pandemic. Identifying the variables that promote or hinder such maintenance will contribute to the development of programs aimed at preventing cognitive decline in healthy older people, as recommended by the American Geriatrics Society^16^.

We aimed to fill this gap by examining neuropsychological measures before and during the pandemic in a group of cognitively healthy older people. Prior to the COVID-19 outbreak, participants had joined the initiatives of an innovative laboratory for active and healthy aging dedicated to training, education, and research at the University of the Third Age (UNITRE) in Turin, Italy. This was the ideal context to identify possible positive responses to the pandemic, in addition to the negative outcomes that have been widely reported (e.g.^17^).

A healthy lifestyle that includes cognitive, social, and physical activities was positively correlated with global cognition^18^, which was achieved through informal learning programs and activities such as the University of the Third Age (U3A)^19^. The combination of positive life experiences such as education and participation in cognitive and socially stimulating leisure activities is thought to increase the effectiveness of cognitive processing in aging individuals, also referred to as cognitive reserve (CR)^20^. It improves cognitive function in healthy aging subjects; in particular, CR has been associated with global cognition^21^ and has been reported to enhance executive function and attention^22,23^. CR has also been shown to be protective against brain damage and dementia^24^, slowing the cognitive aging process or reducing the risk of psychiatric disorders^25^. Indeed, CR can be altered or improved by cognitive, mental, and physical stimulation activities^26^.

Based on these assumptions, participants in our study underwent a thorough neuropsychological assessment before and during the pandemic to identify possible changes in (a) global cognition, memory, language, executive, and attentional functions; (b) physical status; and (c) mood changes. At the final time point (i.e., 21 months after the outbreak of the pandemic), subjects' heart rate variability (HRV), which is an indicator of physiological mechanisms of (dys)regulation^27^, was recorded during the presentation of images depicting the initial and most stressful phase of the pandemic.

We predicted that CR would not only help participants maintain good neuropsychological and physical function to cope with the negative experiences of the pandemic, but also play a protective role in pandemic emotional dysregulation. However, given the potential negative impact of the COVID-19 pandemic, we predicted a possible decline in motivation in the form of apathy and increased anxiety also due to fear of SARS-CoV-2 infection. Moreover, the extent of pandemic-related apathy might reflect the combination of anxiety and emotional (dys)regulation.

## Results

Tables 1 and 2 provide a detailed overview of the characteristics of the 39 participants and their cognitive, affective, and physical status before and during the pandemic. All neuropsychological variables are normally distributed, with no missing data.

**Table 1:** Socio-demographic characteristics and health pandemic-related information of study participants.

	N	%	M	SD
Socio-demographic characteristics				
Subjects	39			
Gender (M/F)	8/31			
Age (years)			70.72	5.62
Education (years)			13.28	2.14
Housing status (single/cohabitant)	16/23			
SES (Hollingshead index)			42.82	8.53
Social stratum 66–55		8%		
Social stratum 54–40		51%		
Social stratum 39–30		36%		
Social stratum 29–20		5%		
Social stratum 19–18		0%		
Information pandemic-related				
Health conditions				
Positivity to COVID-19	1	3%		
Immunization	37	95%		
1st dose	7	18%		
2nd dose	30	77%		
Main preventive measures				
Using face masks		95%		
Wearing latex gloves		87%		
Keeping safety distance		100%		
Washing hands		100%		
Avoiding crowded places		90%		

*N* number, *M* mean, *SD* standard deviation, *M* male, *F* female, *SES* socioeconomic status.

**Table 2:** Demographic and neuropsychological characteristics of study participants before and during the pandemic.

		Before pandemic				During pandemic			Cut-off
		N	M	SD		N	M	SD	
Demographic characteristics									
Subjects		39				39			
Gender (male/female)		8/31				8/31			
Age (years)			67.87	5.76			70.72	5.62	
Education (years)							13.28	2.14	
Neuropsychological assessment									
MMSE			29.23	0.86			29.38	0.77	≥ 23.8
ACE-R			93.28	3.44			94.38	2.86	≥ 79 (< 75 years old);
									≥ 60 (> 75 years old)
MoCA			25.23	2.17			26.36	2.62	≥ 17.363
CFI self-report							2.81	1.72	
CFI partner-report							1.64	1.37	
RMT—15 instant words			44.79	8.92			44.18	7.61	≥ 28.53
RMT—15 delayed words			9.38	2.51			9.56	2.94	≥ 4.69
TMT—part A			38.97	12.09			39.59	13.63	≤ 94
TMT—part B			89.97	24.55			93.74	37.47	≤ 283
TMT B-A			51.01	19.94			54.10	31.90	≤ 187
TT			33.56	2.02			34.79	0.99	≤ 29
Neuropsychiatric assessment									
AES			2.36	3.01			10.97	6.95	≤ 14
BDI			7.33	5.71			7.36	6.50	≤ 9
DIS			1.77	1.79			2.23	1.66	≤ 16.9
HARS			4.97	3.62			8.15	5.16	≤ 14
MAS			1.54	1.99			1.56	1.77	≤ 15
Physical status									
Frailty phenotype			0.33	0.57			0.44	0.63	0
CIRS—severity index			1.25	0.82			1.36	0.19	
CIRS—comorbidity index			0.15	0.67			1.49	0.98	

*N* number, *M* mean, *SD* standard deviation, *MMSE* Mini Mental State Examination, *ACE-R* Addenbrooke’s cognitive examination—revised version, *MoCA* Montreal cognitive assessment, *CFI* cognitive functional instrument, *RMT* Rey memory test, *TMT* trail making test, *TT* token test, *AES* Apathy Evaluation Scale, *BDI* Beck Depression Inventory, *DIS* Disinhibition Scale, *HARS* Hamilton Rating Scale for Anxiety, *MAS* Mania Scale, *CIRS* Cumulative Illness Rating Scale.

Regarding the sociodemographic characteristics of the participants, 79% were women and 21% were men, their mean age was 70 years (age range: 62–82), and their mean educational level was 13 years (range: 8–17). They attended middle school (N = 2), high school (N = 28), and college or university (N = 9).

Forty-one percent of the sample lived alone and the remaining 59% lived with at least one person (in most cases, their partner). According to the Hollingshead index (HI^28,29^), most of the subjects belonged to the medium–high socioeconomic status (SES). Specifically, 8% of them fell into the highest status (HI range: 66–55), 51% into the second (HI range: 54–40), 36% into the third (HI range: 39–30), 5% into the fourth (HI range: 29–20), and none into the lowest (HI range: 19–18).

During the pandemic, only one of the participants had been diagnosed with COVID-19 infection, while the others stated that they had not contracted the virus.

Participants reported high compliance with most of the Italian Ministry of Health recommendations^30^ to contain infection, i.e., maintaining safe distances and frequent hand washing (100% of participants), using face masks (95%), avoiding crowded places (90%), and wearing latex gloves when outdoors (87%).

Regarding immunization against SARS-CoV-2, 95% of participants had been vaccinated against SARS-CoV-2, and 77% had completed two vaccination cycles according to the recommendations of the Italian Ministry of Health.

### Longitudinal analyses of cognitive functioning

Prior to the pandemic, subjects' performance on both the Addenbrooke's Cognitive Examination-Revised Version (ACE-R^31^) and the Mini Mental State Examination (MMSE^32^) did not indicate the presence of mild cognitive impairment (MCI, i.e.^33^), as all subjects scored above cut-off scores and did not report any concerns for subjective cognitive decline (SCD^10^). Their performance was above the reference cut-off on the following tests: the Montreal Cognitive Assessment (MoCA^34^) (100%), Rey Memory Test (RMT^35^)-15 instant words (97%), RMT-15 delayed words (95%), Trail-Making Test (TMT^36^)-part A (100%), TMT-part B (100%), TMT B-A (97%), and Token Test (TT^37^ (100%). Notably, the percentages of results below the cut-off were consistent with the margin of error of the neuropsychological tests in the normative population (see Table 2).

During the pandemic, subjects' performance on both ACE-R and the MMSE did not indicate the presence of MCI (i.e.^24^), as all subjects scored above the regulatory cut-off and did not report SCD^10^, which was also assessed with the Cognitive Function Instrument (CFI^38^). Their performance was above the reference cut-off on the following tests: MoCA (100%), RMT—15 immediate words (97%), RMT—15 delayed words (97%), TMT—Part A (100%), TMT—Part B (100%), TMT B-A (100%), and TT (97%). Again, although some deficits were found in the neuropsychological tests (see Table 2), the percentages of scores below the cut-off were consistent with the margin of error of the tests in the normative population.

To test the stability of cognitive functions, Bayes factors (BFs) were calculated by comparing individual performance before and during the COVID-19 pandemic. In particular, we used the BF with the paired-samples t-test to evaluate the relationship between the probability of the data under the null hypothesis and that under the alternative hypothesis^39–42^. When comparisons were made with BF > 3 (in terms of significantly equal scores), the results showed no longitudinal changes in memory (RMT—15 immediate words, BF_01_ = 4.14; RMT—15 delayed words, BF_01_ = 4.33), attention (TMT time scores: part A, BF_01_ = 5.46; part B, BF_01_ = 3.53; and B-A, BF_01_ = 3.15), and global cognition, as measured by MMSE (BF_01_ = 4.22).

The BF suggests that the data have exactly the same probability of occurrence under the hypothesis that repeated measures are the same, as under the hypothesis that they are different^43^.

We found evidence of differential performance with a significant increase in raw test scores across time points in global cognition, as measured by ACE-R (t = −2.952, p = 0.006, α = 0.05), executive functions (MoCA, t = −2.609, p = 0.013, α = 0.05), and comprehension of linguistic utterances (TT, t = −4.70, p = 0.001, α = 0.05).

### Longitudinal analyses of mood

**Figure 1:**
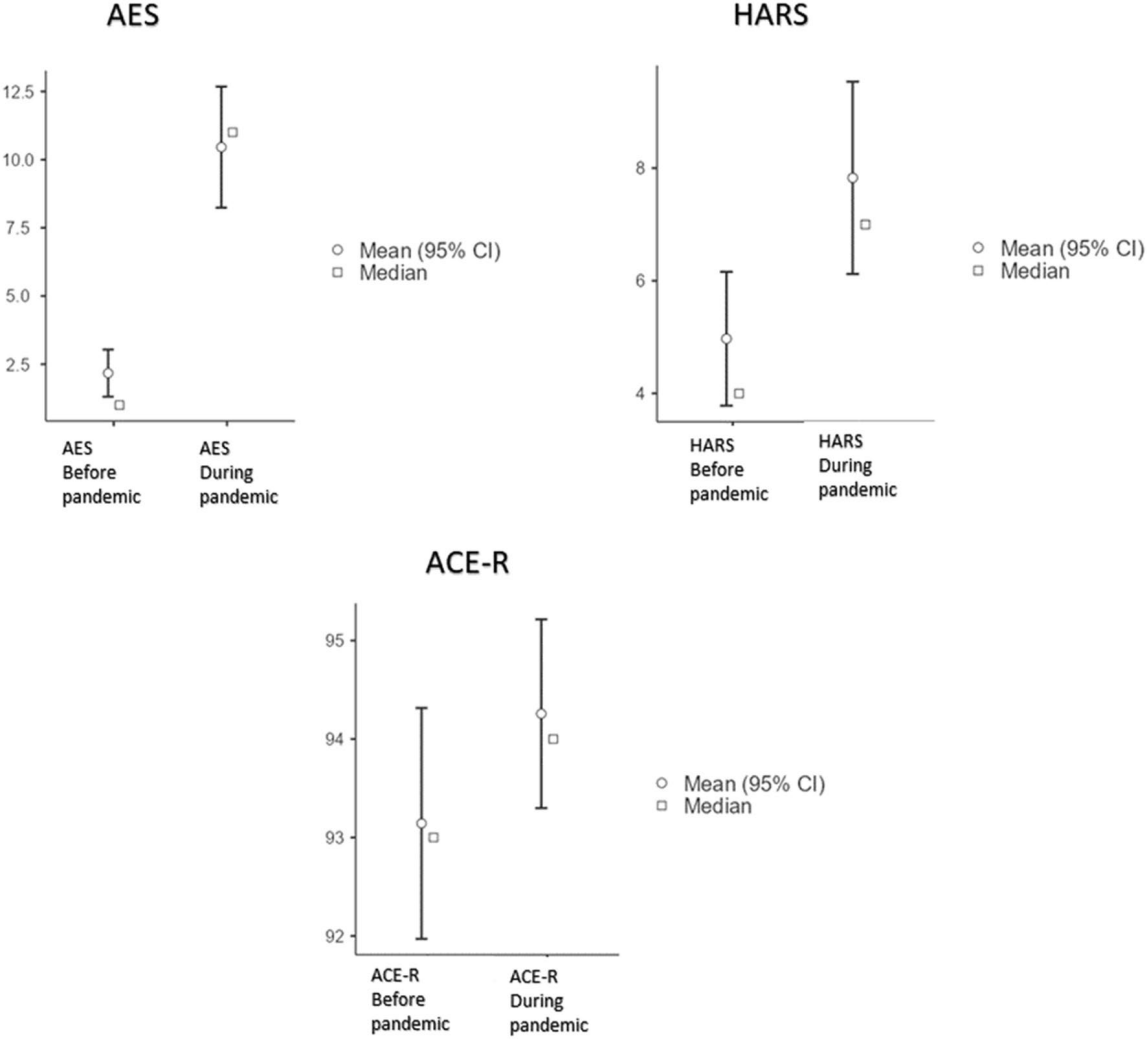
Considering assessments before and during the pandemic, (**a**) the upper part of the figure shows mood changes in terms of apathy (AES, Apathy Evaluation Scale) and anxiety (HARS, Hamilton Rating Scale for Anxiety), and (**b**) the bottom part of the figure shows global cognitive performance on ACE-R (Addenbrooke’s Cognitive Examination—Revised version). For AES and HARS, higher values reflect a more severe symptomatology. For ACE-R, higher values reflect better performance.

The subjects showed longitudinal changes in mood in the form of increasing apathy and anxiety. Prior to the pandemic, none of the subjects scored below the cut-off on the Apathy Rating Scale (AES^44^) and the Hamilton Rating Scale for Anxiety (HARS^45^). Instead, a significant increase in scale scores was observed during the pandemic. Thus, 36% of the sample achieved a below cut-off score on AES and 13% on HARS, respectively. It should be noted that in paired t-tests, there was a significant increase in the mean scores of AES (t = −7.43, p < 0.001, α = 0.05) and HARS (t = −3.71, p < 0.001, α = 0.005), as shown in Fig. 1, which also shows the improvement in ACE-R task performance.

On the other hand, if we consider the comparison with BF > 3 (in terms of significantly equal scores), the results show no longitudinal changes in depression (Beck Depression Inventory, BDI^46^, BF_01_ = 5.33), hypomania (Mania Scale, MAS^47^, BF_01_ = 5.51) and disinhibition (Disinhibition Scale, DIS^48^, BF_01_ = 3.07).

### Longitudinal analyses of physical functioning

As shown in Table 2, most participants were classified as "robust" (72% and 64% before and during the pandemic, respectively); the rest of the sample had a prefrail status (28% and 36% before and during the pandemic, respectively).

None of the participants met Fried et al.'s^14^ inclusion criteria for frailty status, either before or during the pandemic. Based on the McNemar hypothesis test, no changes in physical frailty status were observed (X^2^ = 2.29, p = 0.515, α = 0.05) across timepoints, as shown in contingency Table 3.

**Table 3:** McNemar test.

	Value	df	p
χ^2^	2.29	3	0.515
N	35		

*df* degrees of freedom, *N* sample size.

### Follow-up: correlation matrix and multiple regression analysis

To investigate the relationship between neuropsychological performance at follow-up and emotional (dys)regulation as assessed by the low-frequency/high-frequency (LF/HF) ratio of HRV, we first generated a correlation matrix considering the variables that showed a significant difference before and during the pandemic, based on t-tests and BFs. As shown in Table 4, significant correlations were found between LF/HF and both AES (p < 0.05) and ACE-R (p < 0.05), but not HARS, MoCA, or TT.

**Table 4:** Correlation Matrix—during pandemic.

	AES	LF/HF_ratio	HARS	ACE-R	MOCA
AES	–				
LF/HF_ratio	0.352*	–			
HARS	0.565***	0.053	–		
ACE-R	−0.143	0.364*	0.021	–	
MOCA	−0.022	0.310	0.022	0.712***	–
TT	−0.053	0.072	0.081	0.336*	

*AES* Apathy Evaluation Scale, *LF/HF* low-frequency/high-frequency ratio, *HARS* Hamilton Rating Scale for Anxiety, *ACE-R* Addenbrooke’s cognitive examination—revised version, *MoCA* Montreal cognitive assessment, *TT* token test.

*p < 0.05, **p < 0.01, ***p < 0.001.

AES was selected as the dependent variable of a multiple regression model because it is the measure showing the greatest change across timepoints (t = −7.43, p < 0.001, α = 0.05; Fig. 1). In addition to the LF/HF ratio, which reflects emotional (dys)regulation at follow-up, we modeled as predictors the variables that showed significant change longitudinally, i.e., ACE-R and HARS, which capture global cognitive function and mood anxiety. As shown in Table 5, a strongly significant model (R = 0.711, R^2^ = 0.506) indicated that AES was significantly predicted by lower ACE-R scores (t = −2.391, p = 0.024) and by higher HARS scores (t = 3.704, p = 0.001) and higher LF/HF ratio (t = 3.011, p = 0.006), gender (t = 0.621, p = 0.540) and age (t = −0.630, p = 0.534).

**Table 5:** Model coefficients.

Predictor	Estimate	SE	t	p
Intercept	98.072	44.568	2.201	0.037
ACE-R	−0.968	0.405	−2.391	0.024
HARS	0.688	0.186	3.704	0.001
LF/HF_ratio	3.691	1.226	3.011	0.006
Gender	1.570	2.529	0.621	0.540
Age	−0.131	0.208	−0.630	0.534

Apathy Evaluation Scale (AES) during pandemic as dependent variable of a multiple regression model.

*SE* standard error, *ACE-R* Addenbrooke’s cognitive examination—revised version, *HARS* Hamilton Rating Scale for Anxiety, *LF/HF* low-frequency/high-frequency ratio.

## Discussion

The present study took advantage of the availability of pre-pandemic data on the cognitive, physical, and mental status of a cohort of older people who had participated in the UNITRE Healthy and Active Aging Lab initiatives. The opportunity to directly compare neuropsychological data collected before and during the COVID-19 pandemic, and to investigate possible changes (positive or negative), is unique.

Although the results of some surveys have shown that older people responded better to the COVID-19 pandemic than younger people in terms of emotional well-being, lower reactivity to stressors, and better mental health^49–52^, our study, however, showed for the first time a positive response of healthy older people to the pandemic in terms of neuropsychological performance at different timepoints. Specifically, the data showed (1) significant increases in raw scores on global cognitive ability (ACE-R), executive function (MoCA), and comprehension of linguistic utterances (TT); (2) stable performance on immediate and delayed memory (RMT), executive attention (TMT parts A and B, in addition to TMT B-A), and global cognitive abilities as measured by the MMSE; and (3) stable physical status as measured by phenotypic frailty determinants. Together with the absence of participants classified as "frail," this pattern of findings underscores the maintenance of good physical performance despite inactivity due to the restrictive lockdown measures.

Evidence of stable or even improved measures at different timepoints provides new insight into widely held claims about the negative effects of the pandemic in cognitively preserved older people, based on self-reported observations of deterioration in sleep quality, mental and physical health and functioning, symptoms of depression and apathy, and limitations in social relationships^53^. Although improved performance on attention and working memory tasks has also been reported compared before and during the pandemic measurements, the results of this study were obtained by administering questionnaires adapted for remote administration^53^.

Interestingly, there is evidence that cognitive decline may be mitigated by so-called CR, i.e., the mind/brain's resilience to age- or disease-related changes^54^. Specifically, CR has been shown to mitigate the cognitive^54^ and psychological^55^ effects of physiological aging and, most importantly, its accompanying circumstances such as social isolation and loneliness^3^, which are often cited as major contributors to physical and mental changes during the pandemic^56^. The American Geriatrics Society suggested strategies that could promote healthy aging in the COVID-19 era based on domains that consider health and the promotion of cognitive, physical, and socio-relational aspects^16^.

As reported by WHO^57^, U3A identified as an informal learning program and activity worldwide^19^, and prior to the pandemic, showed a positive impact on the psychological well-being and quality of life of the elderly population. Indeed, U3A programs emphasized improvements in physical health status, emotional balance (e.g., decreases in depressive symptoms and negative affect), social support, and coping activities and strategies, thus increasing positive self-perception and sense of control as key effects for the decade of aging^57^.

Importantly, our results highlight how cognitive functioning can be stimulated and even improved by appropriate interaction with a positive context during the pandemic, as experienced by UNITRE participants who continued to participate in their distance learning courses and shared common experiences via social media. Our study shows that older people can maintain their cognitive levels if they continue to engage in social and educational activities. Moreover, CR appears to play a protective role against the emotional dysregulation associated with the pandemic.

We also observed across different timepoints stable mood scores on the BDI, MAS, and DIS scales, which measure depression, hypomania, and disinhibition, respectively. In contrast, pandemic-related effects appeared to be more associated with mood deflections measured by increased apathy (AES) and anxiety (HARS) during the pandemic.

The results at the correlation matrix, regarding the relationship between neuropsychological follow-up data and emotional (dys)regulation during HRV recording, showed significant associations between the LF/HF ratio and both AES and ACE-R, but not with HARS, MoCA, or TT. In particular, apathy was associated with greater emotional (dys)regulation, and ACE-R scores were related to an increase in the LF/HF ratio, while suggestive pandemic images were presented. Because apathy showed the greatest change across timepoints, it was selected as the dependent variable of a multiple regression model. On this basis, higher apathy was predicted by poorer global cognitive performance, increased anxiety, and emotional (dys)regulation as measured by a higher ratio of low to high frequencies of HRV.

Thus, preserved global cognitive performance appears to play a protective role against the effects of pandemic-related anxiety and emotional dysregulation on apathy.

These findings highlight the importance of CR in attenuating the negative associations between lowered mood and cognition^58^ and their joint contribution to well-being, even in challenging situations such as a pandemic.

## Conclusion

The growing concern triggered by a major health emergency such as the COVID-19 pandemic primarily affects the most vulnerable, for example, the older population with cognitive impairment.

In contrast, our study shows for the first time in the literature how some healthy older subjects responded positively to the pandemic emergency despite some mood deflections, showing *'the bright side of the moon*' through our study. Therefore, the UNITRE model is a good way to promote active and healthy aging, especially in the face of complex and negative events such as the COVID-19 pandemic, even taking into account demographic changes. In fact, by 2050, one in five people will be over 60 years old, which is a total of two billion people worldwide^59^.

Thus, there is an urgent need to plan and implement evidence-based intervention strategies consistent with our study to promote healthy aging that can overcome the adversity of potential future health emergencies. Therefore, UNITRE programs have the potential to accelerate impact by 2023 on healthy aging.

Despite the small sample size, our findings are based on objective measures collected before and during the pandemic through in-depth neuropsychological assessments. This rare opportunity provided new insights into the maintenance of cognitive function and its protective role in relation to changes in mood and emotional (dys)regulation in socially active individuals of moderate to high socioeconomic status.

## Methods

### Participants

From April to October 2019, about 100 socially active older people from the University of the Third Age (UNITRE) in Turin (Italy) joined the initiatives of an innovative laboratory for active and healthy aging, dedicated to training, education and research and development. Before the outbreak of the pandemic, they participated in teaching modules on cognitive functions, physical activity, nutrition and social integration to promote active and healthy aging. Specifically, they were invited to undergo an in-depth neuropsychological assessment to investigate possible associations between cognitive functioning, mood changes, and physical health status over a 3-year period. A group of 81 volunteers (64 women; 60–80 years) agreed to participate in the study, but pandemic restrictions necessitated the establishment of an online assessment. After the lockdown measures were relaxed, 39 subjects agreed to undergo the final date of neuropsychological assessment between June and October 2021. Thirty-five subjects (27 women; 62–82 years) also agreed to participate in a psychophysiological study from October to December 2021 (i.e., 21 months after the pandemic outbreak) to assess emotional reactivity to images associated with the COVID-19 pandemic.

All 39 subjects who participated in the full longitudinal study were > 60 years old and thus can be classified as "older adults"^60^. They were not taking any psychotropic medications that could have affected their cognitive abilities or mood, and none of them complained of cognitive decline^10^. The study was conducted in accordance with the Declaration of Helsinki, having been approved by the Ethics Committee of the University of Turin before (Prot. No. 10038) and during (Prot. No. 151786) the pandemic. All participants gave written informed consent before participating in the study.

### Sociodemographic assessment

In addition to the usual sociodemographic characteristics such as gender, age, and education, we collected participants' socioeconomic status (SES) using the Four Factor Index of Social Status^28,29^, which takes into account educational level, gender, occupation, and marital status. The Hollingshead Index (HI) combines these four factors to assess the social status of individuals or nuclear families. The HI scores can be aggregated into a set of values covering the social strata, ranging from 66 (the highest social stratum: 66–55) to 18 (the lowest social stratum: 19–18). The higher the score, the higher the SES. For example, social stratum 30–39, which reflects the average socioeconomic level, includes skilled craftsmen, clerical, and sales workers.

Sociodemographic characteristics of participants are shown in Table 1, along with information on the pandemic.

The participants were asked whether they had been (a) previously diagnosed with infection by COVID-19 and (b) vaccinated against SARS-CoV-2 (as well as the number of doses administered). In addition, the subjects were asked whether they had followed the recommendations of the Italian Ministry of Health^30^ to control infection, i.e., wearing face masks, wearing latex gloves when outdoors, washing hands frequently, keeping safe distances, and avoiding crowded places.

### Neuropsychological assessment

The study included two time points: before the pandemic, from April to October 2019 (baseline), and during the pandemic, from June to December 2021 (follow-up). To avoid fatigue, a detailed neuropsychological assessment was divided into two sections, lasting approximately 45 min and administered on the same day. The neuropsychological battery was designed to assess three domains, namely cognitive performance, mood, as well as physical and health status.

We assessed global cognitive performance with ACE-R^31^, which includes the MMSE^32^ score. In addition, SCD was assessed before the pandemic using Jessen's criteria^10^ and at follow-up using CFI^38^, which includes both self-report and partner-report to provide a more accurate measure. Executive function was assessed using the MoCA^34^ and the TMT—part A and B^36^. TMT—Part A was used to assess speed of information processing (i.e., psychomotor speed). We assessed language comprehension with TT^37^, while instant and delayed recall were analyzed with RMT^35^.

Different facets of mood were assessed with the following scales: AES^44^, BDI^46^, DIS^48^, HARS^45^, and MAS^47^.

We used the Phenotypic Frailty Model^14^ and the Cumulative Illness Rating Scale (CIRS^61^) to assess physical condition or frailty status and medical history. Five criteria were considered to examine the determinants of a possible physical frailty state: (a) weight loss, (b) grip strength, (c) self-reported fatigue, (d) decreased walking speed, and (e) reduced physical activity. Depending on the presence of the five criteria, participants could be classified as robust (none), prefrail (1 or 2), or frail, (3 or more)^14^.

### COVID-19-related picture stimuli

To assess individual differences in physiological arousal associated with the pandemic, we first searched the "Google images" website (https://images.google.com/) for images showing the most critical period between the virus outbreak and the lockdown in Italy. Using the keyword "COVID”, we obtained an initial selection of 124 images, which were then evaluated separately by all authors to select those that met specific inclusion criteria:

- presence of people regardless of their age, sex, and sociodemographic status in the pandemic context (e.g., healthy individuals, patients, deceased individuals, medical personnel);
- presence of people in the most typical COVID-19 scenarios (e.g., everyday contexts and hospitals).

Based on these criteria, a subset of images was selected for further evaluation based on the following criteria:

- presence of at least 2 people (i.e., "social" images);
- absence of redundant images, i.e., same content but different formats and resolutions (i.e., "unique" images);
- presence of unique signals of the pandemic, such as:
  - masks that had to be worn by at least some of the people depicted;
  - slogans (e.g., "Everything will be fine") and/or banners from balconies;
- the presence of individuals with exclusively Caucasian facial features (i.e., with the same ethnicity as the study participants) to convey greater familiarity and emotional relevance ("ethnicity").

These criteria resulted in a final data set of 75 images randomly presented to each participant during a psychophysiological recording of heart rate variability with self-determined duration. The entire data set had a duration between 45 and 60 min.

### Psychophysiological evaluation

Thirty-five previously studied subjects (27 women; 62–82 years) underwent further psychophysiological recording of HRV while viewing images reminiscent of the initial and most stressful pandemic phase. HRV reflects cardiac autonomic activity (i.e., sympathovagal balance), which indicates the ability of the autonomic nervous system to respond flexibly to external stimuli and to respond to psychophysiological stressors^62^.

The aim of this assessment was to identify possible associations between pandemic-related changes in mood and/or cognitive performance and a proxy for emotional (dys)regulation represented by the ratio of low-frequency to high-frequency HRV, which in turn reflects cardiac sympathovagal balance.

These data were acquired with the Nexus-4 blood volume pulse (BVP) and heartbeat measurement device and subsequently processed with custom-designed software in MATLAB 7.10.0 (R2010a) (The Mathworks, Inc; Natick, MA, USA). Each channel was synchronously recorded at 2048 Hz and extracted at 256 Hz to calculate the indices.

### Signal processing

Cardiovascular and respiratory activities were monitored to assess both voluntary and autonomic effects of breathing on heart rate. The IBI (Inter-Beat-Interval extracted from the BVP sensor, recognized as a measure equivalent to the R-R interval from the electrocardiogram) was analyzed. Following the guidelines of the Task Force of the European Society of Cardiology and the North American Society for Pacing and Electrophysiology, typical indices of spectral HRV were used to assess the autonomic nervous system response^63–67^. Spectral analysis was performed using Fourier spectral methods and dedicated software. The rhythms were classified as very low frequency (VLF, < 0.04 Hz), low frequency (LF, from 0.04 to 0.15 Hz), and high frequency (HF, from 0.15 to 0.5 Hz) oscillations. This procedure allowed us to calculate the LF/HF ratio, also known as the index of sympathovagal balance.

### Statistical analysis

Analyses were performed using Jamovi Statistics software (version 2.2.5.0). Two normality tests (i.e., Kolmogorov–Smirnov and Shapiro–Wilk) were performed to determine whether the variables were normally distributed.

We computed BFs and paired-sample t-tests to compare the data collected before and during the pandemic. The BFs were designed to determine whether variables related to cognition, mood, and physical status remained stable between time points. We tested this hypothesis using the BF, which is a ratio between the probability of the data in the null hypothesis and the alternative hypothesis^39–42^. The evidence for similarity of the measures is considered substantial when 2.5 < BF < 10^39–42^. It is noteworthy that in this case the measures considered are statistically similar, as opposed to the hypothesis that they are different.

BF is generally considered a more reliable statistical test compared to p-value of a t-test. In particular, the American Psychological Association acknowledged the recommendations of the American Statistical Association and emphasized the importance of using BF, among other methods^68^. In this study, we used BF to compare repeated measures and assume similarities or differences between them. Using BF, we were able to determine whether a model that predicted similarities was significantly better than a model that assumed differences. The BF provides the likelihood ratio for this comparison.

To examine the relationship between neuropsychological performance at follow-up and emotional (dys)regulation as indexed by HRV recording, we computed a correlation matrix representing the relationship between (a) the variables associated with a statistically significant difference in the paired t-tests (comparison between before and during the pandemic) and (b) the LF/HF ratio index. The variables that showed a significant difference in the t-tests, in addition to the LF/HF ratio, were then selected as predictors in a multiple regression analysis (adjusted for age and gender), and the neuropsychological scale that showed the greatest change between timepoints was selected as the dependent variable.

## Data Availability

The datasets generated and/or analyzed during the current study are available from the corresponding author on reasonable request.
